# Artificial intelligence in neurocardiology: decoding brain–heart network interactions for clinical and translational insights

**DOI:** 10.3389/fnins.2026.1788653

**Published:** 2026-03-30

**Authors:** Fahimeh Varzideh, Shivangi Pande, Stanislovas S. Jankauskas, Pasquale Mone, Urna Kansakar, Gaetano Santulli

**Affiliations:** 1 Department of Molecular, Cellular and Biomedical Sciences, School of Medicine, City University of New York, Manhattan, NY, United States; 2 Casa di Cura “Clinica Montevergine”, Mercogliano, Italy; 3 International Translational Research and Medical Education (ITME) Consortium, Joint Academic Research Unit, Naples, Italy; 4 Departments of Medicine (Cardiology) and Molecular Pharmacology, Albert Einstein College of Medicine, Wilf Family Cardiovascular Research Institute, Einstein Institute for Neuroimmunology and Inflammation (INI), Fleischer Institute for Diabetes and Metabolism (FIDAM), New York, NY, United States

**Keywords:** AI, autonomic nervous system, brain–heart axis, cardiovascular diseases, deep learning, digital biomarkers, neurocardiology, wearable devices

## Abstract

The intricate interplay between the brain and heart underpins both physiological regulation and pathophysiological processes, yet decoding these interactions remains a formidable challenge. Recent advances in artificial intelligence (AI) offer transformative opportunities to map, model, and predict brain–heart network dynamics with unprecedented precision. This review synthesizes current knowledge on AI approaches applied to neurocardiology, encompassing multimodal data integration from neuroimaging, electrophysiology, autonomic signals, and cardiovascular monitoring. We examine machine learning and deep learning strategies for identifying biomarkers, forecasting adverse cardiac events, and elucidating mechanisms linking neurological, psychiatric, and cardiovascular disorders. Clinical applications are explored across heart failure, arrhythmias, stroke-induced cardiac dysfunction, epilepsy, and stress-related conditions, highlighting AI’s potential for personalized risk stratification. The role of wearable devices, digital phenotyping, and real-world data collection in continuous brain–heart monitoring is discussed, alongside AI-enabled early warning systems. Critical considerations regarding data quality, bias, interpretability, privacy, and ethical governance are emphasized to guide responsible deployment. Finally, we outline emerging directions, including integrative digital twins, federated AI, and closed-loop neuromodulation. By bridging computational innovation and clinical neuroscience, AI-driven approaches promise to redefine neurocardiology, offering predictive, mechanistic, and therapeutic insights into the brain–heart axis.

## Introduction

Understanding the brain–heart connection has evolved from philosophical speculation to rigorous scientific inquiry, revealing a complex bi-directional network that integrates neural, autonomic, humoral, and molecular pathways to regulate the function of the cardiovascular and central nervous systems (Habibi et al., 2026; Saibene et al., 2026; Xu X. et al., 2025). The traditional conceptualization of the heart as merely a pump and the brain as the seat of cognition has been supplanted by a holistic view in which neural circuits continuously influence cardiac physiology and, conversely, cardiovascular signals modulate brain activity (Chen et al., 2025; van Weperen and Vaseghi, 2025). This integrated network, often referred to as the heart–brain axis or brain–heart axis, is mediated primarily by the autonomic nervous system (ANS) and a constellation of cortical and subcortical structures comprising the central autonomic network (CAN), including the insula, hypothalamus, anterior cingulate cortex, amygdala and brainstem nuclei that coordinate sympathetic and parasympathetic outputs to the heart, and receive afferent feedback from cardiovascular sensors (Mura et al., 2026; Valenza et al., 2025).

Physiologically, this axis manifests through multiple communication channels. Neural signaling via sympathetic and parasympathetic fibers adjusts heart rate, contractility, and vascular tone in response to cognitive, emotional, and metabolic demands. At the same time, cardiac afferents convey information about blood pressure, volume, and chemical milieu to brain centers that modulate affective and cognitive states and autonomic output. Humoral and inflammatory mediators, including cytokines, neuropeptides, and microRNAs, also contribute to cross-talk between heart and brain, particularly under conditions of stress or injury, linking systemic inflammation to cardiovascular and neurodegenerative processes (Forte and Casagrande, 2025; Hu et al., 2023; Jia et al., 2025; Roberts et al., 2025). Heart rate variability (HRV) has emerged as a key biomarker of this interconnection, quantifying the oscillatory interplay between sympathetic and parasympathetic inputs and reflecting the integrity of autonomic balance; attenuated HRV is associated with increased risk of cardiovascular mortality, cognitive decline, psychiatric disorders, and adverse outcomes in neurological injury, underscoring the clinical relevance of the brain–heart axis across disease domains (Catrambone et al., 2025; Lyu et al., 2025; MULTi consortium et al., 2025; Prinsen et al., 2025; Reisqs et al., 2025; Wang G. et al., 2025). While specific longitudinal meta-analyses of HRV across neurological and cardiovascular outcomes continue to be published, foundational work has established both prognostic and physiological significance of ANS metrics in integrated brain–heart research (Goh et al., 2025; Hakimjavadi et al., 2025).

Despite advances in physiological characterization, capturing the dynamic complexity and non-linear interactions within the brain–heart network remains challenging with traditional analytic tools. The high dimensionality of physiological signals, temporal dependencies across scales, and context-dependent modulation by behavioral and environmental factors complicate efforts to derive robust mechanistic models or predictive biomarkers. Here, AI and related computational approaches offer novel opportunities. AI encompasses a broad set of techniques, including machine learning (ML), deep learning (DL), and advanced signal processing, that can extract latent patterns from large, multimodal datasets, integrate heterogeneous inputs (including EEG, ECG, HRV, imaging), and potentially infer latent interaction patterns beyond linear associations.

AI methods have already demonstrated substantial promise in individual organ system applications (Table 1). For example, DL models such as convolutional neural networks (CNNs) and hybrid architectures have been used to automatically classify cardiac arrhythmias from ECG signals with high accuracy and reliability, outperforming traditional signal-processing methods and offering potential real-time diagnostic capabilities (Bartusik-Aebisher et al., 2025; Hannun et al., 2019). These successes illustrate how AI can learn complex temporal features and non-linear relationships from high-dimensional biosignals, underpinning its applicability to integrated brain–heart datasets where standard statistical modeling is insufficient.

**TABLE 1:** AI approaches applied to brain–heart or closely related physiological data.

AI/ML method	Input data type	Typical application(s) in brain–heart axis research	Key strengths	Evidence
Convolutional neural networks (CNNs)	Single-lead or multi-lead ECG, wearable ECG, PPG	Arrhythmia detection (AFib, VT), subclinical cardiac disease, autonomic proxies	Automatic feature learning from raw signals; high accuracy in waveform morphology	Translational → Clinical (cardiac-focused)
Recurrent neural networks (LSTM/GRU)	Sequential ECG, HRV time series, EEG–ECG sequences	Rhythm classification, temporal dependency modeling, stress recovery, seizure-related autonomic changes	Captures long-range temporal structure and delayed neurocardiac effects	Experimental → Translational
Hybrid CNN–RNN models	ECG + HRV, EEG–ECG	Multiclass arrhythmia classification, neurocardiac coupling	Combines spatial feature extraction with temporal dynamics	Translational
Random forests	Hand-engineered HRV features, ECG-derived metrics, clinical metadata	Cardiovascular risk stratification, autonomic dysfunction assessment	Robust to noise; interpretable feature importance; good baseline performance	Experimental → Translational
Support vector machines	ECG/HRV features, EEG spectral features	Stress detection, autonomic state classification, cognitive load estimation	Effective in small, high-dimensional datasets; well-characterized behavior	Experimental
Autoencoders (AEs/VAEs)	ECG or EEG time series	Unsupervised anomaly detection, denoising, latent state discovery	Label-free representation learning; useful for rare-event detection	Experimental
Attention-based DL Models	Multimodal EEG–ECG, longitudinal wearable data	Stress detection, outcome prediction, workload assessment	Dynamic weighting of temporally relevant segments	Experimental → Translational
Multimodal DL (fusion models)	EEG, ECG, HRV, imaging, wearable data	Post–cardiac arrest prognosis, stroke-related cardiac risk, affective disorders	Captures cross-modal synergy beyond single-modality models	Translational
Graph neural networks (GNNs)	Brain connectivity (EEG/fMRI), HRV networks	Modeling CAN connectivity, network-level autonomic dysregulation	Explicit representation of physiological networks	Experimental
Generative models (GANs, VAEs)	ECG, EEG	Data augmentation, synthetic physiology generation	Mitigates data scarcity and class imbalance	Experimental
Causal/explainable AI	Multimodal time-series (EEG–ECG–HRV)	Directionality inference, mechanistic insight	Improves interpretability and hypothesis generation	Experimental
Digital twin/closed-loop AI	Continuous wearable + clinical data	Personalized risk forecasting, adaptive neuromodulation	Individualized, dynamic modeling	Conceptual/Early Experimental

The integration of AI into the study of brain–heart interactions thus promises to transcend conventional statistical analyses by enabling *data-driven discovery* of interdependent features that reflect both neural activity and cardiovascular function, fostering predictive models of disease progression, risk stratification, and therapeutic response. For example, AI can combine continuous physiological signals from wearable devices with neurophysiological markers to detect early autonomic dysfunction, predict acute events such as stroke or myocardial infarction, or monitor recovery trajectories after neurologic injury. Coupled with real-time data streams and longitudinal monitoring, such systems could support personalized, adaptive healthcare paradigms that continuously assess an individual’s neural–cardiac state and alert clinicians to destabilizing trends (Amador et al., 2025; Gilani et al., 2025; Perez Vicencio et al., 2026).

Furthermore, advances in neuroimaging and multimodal data fusion (integrating structural and functional MRI, electrophysiological recordings, and peripheral biomarkers) expand the scope of AI applications to include mechanistic mapping of CAN connectivity, delineation of neural circuits modulating cardiac function, and adaptive models that account for individual variation in physiology and pathology. By embracing AI approaches that can handle high-dimensional, time-series data, researchers can begin to unpack the non-linear, context-dependent relationships underpinning disease states where the brain–heart axis is perturbed, such as in heart failure, stress cardiomyopathy, epilepsy, and stroke-associated cardiac dysfunction (Fan et al., 2025; Fu et al., 2026; Hu et al., 2023; Johnston et al., 2025).

Despite this potential, there are significant methodological and translational challenges, including standardizing data acquisition, ensuring model interpretability, addressing bias and privacy concerns, and validating AI models across diverse clinical populations. Nevertheless, the synergy between computational innovation and an increasingly sophisticated understanding of the brain–heart axis positions AI as a transformative tool in decoding physiological complexity and advancing integrated neurocardiac science (Abohashem et al., 2025; Grifoni et al., 2025; Kirkpatrick, 2022; Li Q. et al., 2025; Wu et al., 2025).

## Data sources for AI-based brain–heart analysis

Research into AI-based decoding of brain–heart network interactions draws from increasingly rich and complex datasets that capture both neural and cardiovascular physiology (Table 2). To model the multi-directional communication between the brain and the heart, investigators rely on multimodal signals, including neurophysiological recordings, cardiac electrophysiology, imaging, wearable biosensors, and clinical metadata. Each data source brings unique insights and analytic challenges, and AI methods are essential for extracting meaningful patterns from this high-dimensional information (Fernández-Avilés, 2025; Haque and Dutta, 2026; Palantzas and Anagnostouli, 2025; Toyli et al., 2025).

**TABLE 2:** Clinical applications of AI relevant to brain–heart interactions.

Clinical domain	Example AI application	Typical data inputs	Summary of findings	References
Cardiovascular	AFib detection using wearable ECG	Smartwatch ECG	High sensitivity and specificity for AFib screening	(Perez et al., 2019)
Cardiovascular	ECG-based prediction of future AFib	12-lead ECG	AI identifies latent atrial cardiomyopathy	(Baek et al., 2023)
Neurocardiology	Autonomic dysfunction after stroke	ECG, HRV	Stroke-related autonomic imbalance predicts mortality	(Ornello et al., 2018)
Neurology	Markers of Sudden Unexpected Death in Epilepsy (SUDEP)	ECG, EEG	Cardiac autonomic markers linked to seizure-related death	(Lagae et al., 2016)
Mental health	Stress detection from wearables	HRV, activity	ML distinguishes stress from baseline physiology	(Lagae et al., 2016)

### Neurophysiological data

Neurophysiological signals such as electroencephalography (EEG), magnetoencephalography (MEG), and neuroimaging (fMRI, PET) provide direct measurements of brain activity across spatial and temporal scales. EEG and MEG are particularly valuable for capturing fast electrical dynamics related to cognitive states, autonomic regulation, and emotional processing (all domains that intersect with cardiovascular control). Conventional analysis techniques like frequency band power or connectivity metrics are limited when faced with the complex, non-linear dynamics characteristic of brain–heart coupling, motivating the adoption of ML and DL to capture latent dependencies in high-dimensional neural time series (Carlén et al., 2025; Hansen et al., 2025; Sayed et al., 2025; Tardo et al., 2025).

EEG has been used in AI frameworks to predict clinical outcomes when combined with cardiac data. ML models integrating EEG, ECG, and clinical features achieve excellent predictive performance for neurological outcomes after cardiac arrest, reaching area under the curve values near 1.0 during early critical time windows (Zheng et al., 2022; Zubler and Tzovara, 2023). This observation underscores the advantage of combining neural and cardiac signals with AI to improve prognostic accuracy beyond what either modality could achieve alone.

### Cardiac and vascular data

Electrocardiography (ECG) is the cornerstone of cardiac electrophysiology and provides high-fidelity information about rhythm, conduction, and autonomic influences such as HRV. AI applied to ECG data has been one of the most successful and clinically impactful subfields in cardiovascular AI research (Battiloro and Dominici, 2025; Hempel et al., 2025; Herman and Smith, 2025; Lee H. et al., 2025; Nakamura and Sasano, 2025; Nash et al., 2025; Ribeiro and Ribeiro, 2025). AI algorithms, including CNNs and recurrent architectures, outperform traditional methods in diagnosing arrhythmias, heart failure, and myocardial infarction, and can detect subclinical disease features previously undetectable with standard interpretation (May and Kashou, 2024; Tokodi and Kovacs, 2024; Velandia et al., 2025).

Wearable devices with ECG capability have accelerated the generation of large-scale, real-world cardiac datasets. DL algorithms applied to smartwatch ECG recordings can identify conditions like atrial fibrillation (AFib) and prolonged QT syndrome with promising accuracy. These large, heterogeneous, and longitudinal data are ideally suited for AI models that learn complex patterns over time (Armoundas, 2025; Bartusik-Aebisher et al., 2025; Devkota et al., 2025; Herman et al., 2025; Lewontin et al., 2025; Lopez-Jimenez et al., 2025; Love et al., 2025; Magnani et al., 2025; Suzuki et al., 2026; Thomas et al., 2025; Yoon and You, 2025).

In brain–heart research specifically, ECG serves not only as a marker of cardiac health but also as a surrogate indicator of autonomic state related to brain processes such as stress, cognition, and emotional regulation. Multimodal AI frameworks increasingly treat ECG features (like HRV metrics) as essential inputs for models that quantify physiological coupling between heart and brain signals, enabling more robust predictions of outcomes or states influenced by both systems (Bahameish et al., 2024; Godwin et al., 2024; Jang et al., 2021).

### Multimodal and longitudinal datasets

The defining strength of AI in brain–heart analysis lies in its ability to integrate multimodal datasets, which can include simultaneous EEG and ECG recordings, imaging data, wearable sensor outputs, and clinical metadata such as demographics, risk factors, and outcomes (Frey et al., 2026; Zhou et al., 2026). Multimodal AI techniques (such as early fusion, intermediate representation learning, and hybrid models) enable algorithms to learn complementary features across modalities rather than treating each signal in isolation (He et al., 2026; Zhang R. et al., 2025). This approach has been shown to enhance predictive accuracy and capture richer representations of physiological state than single-modality models alone. For instance, multimodal integration of imaging, electrophysiology, and clinical features has been shown to significantly boost model performance in cardiovascular applications (Candia-Rivera et al., 2025; Yang et al., 2025).

Beyond health systems and clinical trials, wearable data streams now generate continuous, real-world physiological datasets that provide unprecedented granularity and breadth (Table 3). Longitudinal ambulatory ECG, photoplethysmography (PPG), and emerging wearable EEG devices offer opportunities for dynamic AI monitoring of brain–heart interactions outside controlled environments, although challenges remain in harmonization and noise mitigation for robust model training (Alzghaibi, 2025a; Alzghaibi, 2025b; Barua et al., 2025; James et al., 2025; Klein et al., 2025; Mahajan et al., 2025; Olawade et al., 2025; Radanliev, 2025; Tripathi et al., 2021; Wang J. et al., 2025).

**TABLE 3:** Wearables and digital phenotyping studies using AI.

Study	Wearable type	Modalities	Analytic approach	Key outcome	References
Apple heart study	Apple Watch	ECG	CNN-based rhythm classification	Population-scale AFib screening	(Perez et al., 2019)
Depression and sleep phenotyping	Actigraphy + HRV	Activity, HRV	Statistical & ML modeling	Physiological markers associate with depression	(Song et al., 2023)
Stress detection	Wearable sensors	HRV, motion	Supervised ML	Accurate stress state classification	(Basem et al., 2025)
AFib detection validation	Patch ECG	ECG	Deep learning	Comparable to cardiologist interpretation	(Hannun et al., 2019)

These data sources illustrate the diverse landscape of signals available for AI-driven brain–heart interaction research. While traditional approaches often analyze individual modalities separately, AI methods facilitate integrated, multimodal analyses that capture the underlying complexity of physiological networks. In the next section, we will explore the specific AI methodologies that enable these advances, focusing on how they handle high-dimensionality, non-linearity, and temporal dynamics in brain–heart datasets.

## AI Methodologies for decoding brain–heart network interactions

The study of brain–heart network interactions demands analytic approaches capable of handling *complex, non-linear, multimodal*, and *high-dimensional* physiological data. AI provides a versatile set of tools that extend beyond classical statistical techniques, enabling researchers to uncover latent structure, cross-modal dependencies, temporal dynamics, and predictive biomarkers throughout the neurocardiac axis (Bhatti et al., 2025; Mehmood et al., 2025).

### ML foundations in neurocardiac modeling

Traditional supervised ML has been widely used as a first step toward AI-assisted physiological modeling. In this context, ML algorithms operate on *hand-engineered features* extracted from cardiac and neural signals, such as HRV metrics, spectral power from EEG, and time-domain characteristics of ECG. Algorithms like *support vector machines* (Scattone et al., 2025; Srivastava et al., 2025; Wanyonyi et al., 2025), *random forests* (Wanyonyi et al., 2025; Xu Z. et al., 2025), and *gradient boosting* (Innab et al., 2024; Lee et al., 2022) have been used to classify states associated with autonomic balance, stress responses, and arrhythmia risk across populations with autonomic dysfunction or cognitive stress (Ashtiyani et al., 2018; Jaya Prakash et al., 2025).

In cardiovascular research, ML and shallow neural models, when combined with expert-derived features, can outperform traditional rule-based criteria but are generally surpassed by DL in performance and robustness (Albarrak et al., 2025).

Although feature engineering allows physiologically interpretable inputs, it is limited by human expertise and may miss complex signal interactions inherent to brain–heart coupling. This limitation motivates the shift toward *DL*, which automatically learns hierarchical representations from raw or minimally processed data.

### DL Architectures for physiological time series

DL has rapidly become the dominant AI paradigm for processing physiological data, particularly in ECG analysis, but also EEG and combined neurocardiac signals. DL architectures such as CNNs, recurrent neural networks (RNNs), and hybrid networks including Long Short-Term Memory (LSTM) and Gated Recurrent Unit (GRU) units are capable of *end-to-end representation learning*, bypassing the need for handcrafted features (Pokaprakarn et al., 2022). In cardiology, a landmark example demonstrated that a deep neural network trained on > 2 million 12-lead ECGs could automatically detect multiple abnormalities with performance exceeding that of resident cardiologists, illustrating the power of DL for complex cardiac signal interpretation (Ribeiro et al., 2020).

CNNs excel at capturing local spatial and morphological patterns within raw ECG waveforms, enabling them to distinguish subtle deviations in the cardiac cycle that may escape classical algorithms. CNN-dominant models consistently achieve high accuracy (> 95% in many tasks), outperform traditional ML and shallow models on public benchmark datasets (Banta et al., 2021; Saglietto et al., 2024).

Time dependency between neural and cardiac signals, such as prolonged cardiac intervals in response to stress or neurological state changes, is effectively modeled by recurrent architectures (LSTM, GRU). Hybrid CNN-LSTM models combine spatial feature extraction with temporal pattern learning, allowing robust electrocardiographic arrhythmia classification and prediction that captures both beat morphology and sequence context. Hybrid DL models have achieved high performance in arrhythmia classification across multiple classes and are among the more popular architectures in physiological time-series analysis (Najia and Faouzi, 2025).

Recent advances include leveraging attention mechanisms on top of CNN and RNN modules to allow models to *dynamically weight different temporal segments*, which is especially useful when signals from different modalities (e.g., EEG and ECG) demonstrate varying relevance across states like stress or recovery.

### Multimodal integration and deep representation learning

Brain–heart interactions are *multimodal by nature*, involving concurrent dynamics in neural and cardiovascular domains. AI methods that *integrate across modalities* have shown improved performance in classification and prediction tasks relative to single-modality models, as they can learn synergistic patterns that emerge only in joint analysis (Mura et al., 2026; Shi et al., 2025; van Weperen and Vaseghi, 2025).

Multimodal fusion strategies fall into three broad categories: (1) *Early fusion*, where raw signals or precomputed features from EEG, ECG, and possibly imaging are concatenated before training; (2) *Intermediate fusion*, where modality-specific networks learn latent representations that are then combined; (3) *Late fusion*, where separate modality-specific models produce predictions that are aggregated.

Across physiological domains, evidence suggests that intermediate fusion often provides the best balance between modality-specific learning and cross-modal synergy, enabling models to learn complex interdependencies without overwhelming early layers with heterogeneous signal formats. Models combining neural and cardiac data outperform unimodal systems in tasks such as stress detection and workload classification (Pataca et al., 2025; Wang and Wang, 2025).

### Graph neural networks and network-level modeling

Given the network character of brain–heart systems, representing physiological interactions as graphs allows AI models to explicitly reason over *connectivity patterns* rather than isolated channels. Graph neural networks (GNNs) propagate information across nodes representing brain regions or cardiac features and edges encoding functional or structural links.

GNNs have been used to model brain connectivity in EEG and fMRI, while recent studies in cardiovascular research apply graph reasoning to HRV and ECG feature networks to understand autonomic regulation patterns (Liu S. et al., 2025; Roovers et al., 2024). By integrating neural and cardiac nodes into joint graphs, GNNs can learn how perturbations in one domain propagate to the other, offering a promising avenue for capturing *clinically relevant network disruptions* such as those seen in heart failure or post-stroke autonomic dysfunction (Cai et al., 2022; Saini and Rose, 2025).

### Generative models, data augmentation, and synthetic physiology

Deep generative models such as generative adversarial networks (GANs), variational autoencoders (VAEs), and *diffusion models* are gaining traction in physiological signal analysis, offering robust methods for data augmentation, denoising, and synthetic data generation. Generative models have been successfully applied to ECG and EEG time series for tasks such as noise reduction and training data expansion, addressing the common challenge of limited labeled data in medical datasets (Neifar et al., 2025).

By generating realistic synthetic signals, generative models improve model robustness and help mitigate biases due to imbalanced classes (like rare arrhythmias or atypical EEG patterns), which is particularly valuable for brain–heart integrative studies where simultaneous neural and cardiac events may be rare (Simone et al., 2025).

### Causal inference, temporal dynamics, and explainability

Predictive accuracy alone is not sufficient for mechanistic understanding or clinical translation. Contemporary AI research emphasizes *causal inference* and dynamic modeling to quantify directionality and interactions between physiological systems. Tools such as causal neural networks, temporal attention models, and Granger causality-inspired architectures aim to reveal whether changes in neural activity drive cardiac outcomes or whether cardiac feedback modulates neural states (Jiao et al., 2024).

Additionally, explainable AI (XAI) techniques are essential to elucidate model decisions, particularly in clinical settings where interpretability influences trust and adoption. Methods such as saliency maps, SHAP values, and attention visualization help highlight which portions of EEG or ECG data contribute most to predictions, and can surface novel *mechanistic hypotheses* about brain–heart coupling (Abbas et al., 2025).

## Clinical and translational applications of AI-decoded brain–heart interactions

The integration of AI into the evaluation of brain–heart network interactions is rapidly transitioning from a conceptual framework to clinically relevant and translational applications. By enabling the joint analysis of neural and cardiovascular data, AI models provide new opportunities for early diagnosis, risk stratification, therapeutic monitoring, and personalized medicine across a range of neurological and cardiovascular disorders (Jankauskas et al., 2026).

One of the most direct clinical applications of AI-based brain–heart modeling lies in the assessment of ANS dysfunction, a common pathophysiological substrate linking neurological and cardiovascular disease. Alterations in central autonomic network activity (encompassing regions such as the insula, anterior cingulate cortex, and brainstem nuclei) have measurable downstream effects on cardiac electrophysiology, particularly HRV and arrhythmic susceptibility (Basem et al., 2025).

AI models that integrate EEG-derived neural features with ECG-based autonomic markers have demonstrated improved performance in identifying individuals at elevated cardiovascular risk compared with cardiac markers alone. HRV-based ML and DL models have been shown to predict mortality and major adverse cardiovascular events with high accuracy, reflecting impaired neurocardiac regulation (Zillner et al., 2024).

Importantly, AI frameworks that incorporate neural inputs move beyond peripheral markers, enabling earlier detection of dysautonomia before overt cardiac pathology develops. This approach has particular relevance for populations with diabetes, neurodegenerative disease, or chronic stress exposure, in whom autonomic imbalance precedes clinical cardiovascular events.

### Neurological disorders with cardiac sequelae

AI-decoded brain–heart interactions are increasingly relevant in neurological disorders where cardiac complications contribute substantially to morbidity and mortality. Stroke, epilepsy, traumatic brain injury, and neurodegenerative diseases all disrupt central autonomic control, predisposing patients to arrhythmias, myocardial injury, and sudden cardiac death (Ornello et al., 2018; Rezk et al., 2025).

In acute ischemic stroke, alterations in insular cortex activity have been linked to malignant arrhythmias and adverse outcomes. AI models integrating neuroimaging, EEG, and ECG data have shown promise in predicting post-stroke cardiac complications. Autonomic and cardiac abnormalities following stroke are strongly associated with lesion location and neural network disruption, reinforcing the importance of brain–heart coupling in post-stroke prognosis (Fan et al., 2024).

Similarly, in epilepsy, AI analysis of combined EEG–ECG datasets has improved prediction of seizure-related cardiac dysregulation, a key factor implicated in sudden unexpected death in epilepsy. DL models have been used to detect pre-ictal autonomic changes, suggesting potential for real-time monitoring and early intervention (Lagae et al., 2016; Plummer et al., 2019).

### Mental health, stress, and affective disorders

Mental health disorders represent a major translational frontier for AI-based brain–heart research. Depression, anxiety, and chronic stress are associated with persistent autonomic imbalance, inflammation, and elevated cardiovascular risk. Traditional psychiatric assessment relies heavily on subjective reporting, whereas AI-decoded brain–heart metrics offer objective physiological biomarkers.

Multimodal AI models combining EEG markers of emotional processing with ECG-derived HRV features have been shown to accurately classify stress states and depressive symptom severity. Reduced HRV and altered prefrontal EEG activity jointly predict major depressive disorder and cardiovascular risk, supporting a neurocardiac model of affective illness (Galin and Keren, 2024; Lazarou and Exarchos, 2024; Zhou et al., 2019).

These approaches are particularly relevant for precision psychiatry, where AI-derived neurocardiac signatures could guide individualized treatment selection, monitor therapeutic response, and identify patients at heightened cardiometabolic risk.

### Perioperative and critical care monitoring

In perioperative and critical care settings, AI-based brain–heart monitoring holds significant promise for early deterioration detection. Continuous EEG and ECG monitoring generates vast amounts of data that exceed human interpretive capacity, making them ideal targets for AI automation.

DL models integrating neural and cardiac signals have demonstrated high accuracy in predicting neurological outcomes after cardiac arrest and in identifying early signs of autonomic collapse in intensive care units. ML models using EEG and ECG features could accurately predict poor neurological outcomes within hours after resuscitation, outperforming conventional prognostic tools (Kawai et al., 2025; Niu et al., 2025; Xu et al., 2026). Such tools could enable proactive intervention, optimize resource allocation, and improve survival and functional outcomes in high-risk patients.

### Wearables, remote monitoring, and preventive medicine

The rapid expansion of wearable technologies has created unprecedented opportunities for AI-driven, real-world brain–heart monitoring. Smartwatches, chest patches, and emerging wearable EEG devices continuously collect physiological data across daily life, enabling longitudinal assessment of neurocardiac dynamics. AI algorithms applied to wearable ECG data have already demonstrated clinical utility in detecting AFib and autonomic dysfunction at population scale (Abedi et al., 2025). The next translational step involves integrating neural proxies (such as sleep EEG or surrogate cognitive markers) to capture brain–heart interactions outside clinical environments.

This paradigm supports a shift from reactive to preventive cardiovascular and neurological care, enabling early identification of dysregulation and timely intervention before irreversible disease develops.

Collectively, these applications illustrate how AI-decoded brain–heart interactions are moving toward clinical implementation across cardiology, neurology, psychiatry, and critical care (Dang et al., 2021; Dangi et al., 2025; Lee G. et al., 2025; Trasca et al., 2025; Zhang H. et al., 2025). While challenges remain in validation, interpretability, and integration into clinical workflows, the translational potential of this field is substantial. In the next section, we will address the current limitations, ethical considerations, and future directions necessary to ensure safe, equitable, and effective deployment of AI-based brain–heart technologies.

## Clinical applications

### Cardiovascular diseases

AI and wearable monitoring have begun to reshape cardiovascular diagnostics and management, with direct implications for brain–heart interactions.

AI-assisted ECG analysis using wearables and ambulatory data has shown high accuracy in detecting arrhythmias such as AFib and ventricular arrhythmias, which often involve autonomic dysregulation linked to central neural circuits (Baek et al., 2023; Perez et al., 2019). DL models have achieved performance approaching cardiologist-level accuracy in rhythm classification from single-lead ambulatory ECG, enabling population-scale detection of silent arrhythmias that might otherwise go unnoticed until adverse events occur.

Beyond rhythm classification, AI-ECG models applied to standard and wearable ECGs are advancing detection of heart failure, structural abnormalities, and arrhythmic substrates, with performance metrics suggesting earlier and more accurate disease identification than conventional ECG interpretation (Accorsi et al., 2025; Alexandrino et al., 2025; Ameziane et al., 2024; de Alencar and Smith, 2025; Gupta et al., 2025; Huang X. et al., 2025; Lee S. et al., 2025; Oikonomou, 2025; Pavluk et al., 2026; Velandia et al., 2025; Yisimitila et al., 2025).

AI applied to wearable ECG and photoplethysmography (PPG) devices is also being explored for predictive analytics, such as forecasting incident hypertension (a key cardiovascular risk factor with implications for brain perfusion and neurovascular coupling) outperforming traditional risk stratifiers (Varzideh et al., 2026). For example, the AIRE-HTN AI-ECG model discriminated future hypertension risk and stratified hypertensive progression above established clinical predictors in two cohort datasets (Sau et al., 2025).

Wearable technology also enhances continuous HRV and arrhythmia monitoring in perioperative and postoperative populations; early detection of post-operative AFib (POAF) via wearable HRV monitoring could reduce stroke and heart failure risk by enabling prompt interventions (Zillner et al., 2024).

Taken together, these applications illustrate how wearables combined with AI can provide early detection, real-time risk assessment, and ongoing monitoring of cardiologic conditions, many of which involve autonomic disturbances mediated by central neural processes.

### Neurological disorders

Neurological disorders often present with secondary cardiac dysfunction through autonomic dysregulation and disrupted brain–heart communication.

While few AI studies have directly targeted brain–heart interactions in neurological disorders, wearable and AI-enhanced HRV assessments are being used to probe autonomic dysfunction in conditions such as Parkinson’s disease, where HRV signatures captured by wearable ECG correlate with clinical features and may reflect underlying neurodegenerative changes in autonomic circuits (Park et al., 2025).

In epilepsy, cardiorespiratory and autonomic dynamics during seizures can precipitate arrhythmias or changes in heart rate patterns. Although direct AI models for predicting cardiac dysfunction from EEG/ECG in epilepsy are emerging in preprint/formative research (like cross-modal ECG/EEG cognitive load models or EEG surrogates from ECG), validation in clinical cohorts is an active area of research (Kalousios et al., 2025; Li Y. et al., 2025).

Stroke survivors are also at elevated risk of cardiac arrhythmias and myocardial injury due to autonomic imbalance and neurogenic effects. AI tools capable of integrating ambulatory cardiac data with clinical and neuroimaging features could help identify high-risk patients, although rigorous prospective studies are still needed.

### Mental health and stress-related conditions

Changes in HRV and wearable-derived physiological patterns have been linked to mental health states such as anxiety, depression, and chronic stress, all of which carry elevated cardiovascular risk and are mediated by central autonomic regulation.

Remote digital HRV measurement via wearables provides a digital biomarker of autonomic balance, reflecting sympathetic and parasympathetic activity. Contemporary literature highlights its utility in monitoring stress responses and predicting changes in mental health severity (for instance depression and anxiety), which may in turn inform cardiovascular risk profiles (Owens, 2020). Moreover, wearable sensor data analyzed with AI (including HRV, physical activity, and skin conductance) serve as digital phenotypes of stress and emotional states that correlate with mental health diagnoses and may also pre dysregulation if chronic sympathetic overactivation persists (Kargarandehkordi et al., 2025). These applications are foundational for future brain–heart models that combine physiological signals with cognitive and emotional biomarkers, with the aim of detecting risk patterns that span psychiatric and cardiovascular domains.

## Wearables, digital phenotyping, and real-world AI

### Continuous brain–heart monitoring

Wearables capable of continuous physiological monitoring (including ECG, PPG-derived HRV, and ambulatory activity) provide unique data to model real-world neurocardiac dynamics.

Continuous monitoring of HRV and arrhythmias via wearable ECG and PPG sensors has demonstrated value in detecting clinically relevant events that intermittent monitoring would miss, particularly in cardio-surgical and ambulatory contexts (Zillner et al., 2024). HRV in these devices reflects autonomic state and, by extension, neurovisceral integration. HRV wearable monitoring captures fluctuations in sympathovagal balance during daily life and stress, which are correlates of both mental and cardiovascular health (Alugubelli et al., 2022; Zillner et al., 2024).

These continuous signals provide the substrate for real-time AI analysis, enabling detection of transient events like arrhythmias and autonomic shifts, as well as longer-term trends that may precede clinical deterioration.

### Passive data collection and digital biomarkers

Passive collection of physiological data such as HRV, sleep patterns, physical activity, and skin conductance forms a digital biomarker battery indicative of an individual’s health state. Such proxies go beyond traditional episodic measures and can reflect cumulative autonomic load and behavioral phenotypes (Grosicki et al., 2026; Song et al., 2023).

AI models applied to wearable data have successfully predicted mental health states, stress levels, and cognitive function indices, suggesting that digital biomarkers can capture aspects of neurocardiac regulation in everyday contexts (Hussain et al., 2026; Kargarandehkordi et al., 2025).

Importantly, these digital biomarkers require rigorous validation and contextual calibration, as wearable sensors vary in accuracy and are influenced by movement, sensor placement, and environmental factors.

### AI-enabled early warning systems

The combination of continuous data streams with AI analytics supports early warning systems that detect deviations from individualized baselines and forecast adverse events before they manifest clinically. AI applied to wearable ECG data has been proposed for automated population-scale screening and early detection of arrhythmias and structural abnormalities, enabling timely intervention and management (Bartusik-Aebisher et al., 2025). Similarly, real-time detection of stress-related ANS imbalance via wearable HRV and PPG could permit early behavioral or therapeutic adjustments to mitigate downstream cardiovascular risk (Kargarandehkordi et al., 2025; Tsai et al., 2025).

These systems exemplify how real-world AI can bridge continuous physiological monitoring with actionable prognostics, with the caveat that large-scale prospective validation and integration into clinical workflows remain necessary for widespread clinical implementation.

## Validation, bias, and ethical considerations

### Data quality, labeling, and harmonization

One of the fundamental challenges in applying AI to healthcare (including multimodal brain–heart signal analysis) is ensuring data quality and harmonization across sources. Medical data are typically collected from electronic health records (EHRs), diagnostic devices, imaging, wearables, and research cohorts. These sources use heterogeneous formats, sampling rates, sensor types, and labeling conventions, complicating their integration for AI training (Stark et al., 2025). Inconsistent annotation practices and missing data adversely affect the reliability and reproducibility of learned patterns, especially in complex neural and cardiac signals where precise temporal alignment is critical. Poor data quality amplifies model errors and undermines clinical trust (Acosta et al., 2022; Fernandes Prabhu et al., 2025).

Addressing these issues requires standardization frameworks, common ontologies, robust labeling pipelines, and curated multicenter datasets (Bakken, 2024). Additionally, rigorous cross-validation and external validation using independent cohorts are essential for demonstrating that AI models perform consistently across clinical environments rather than simply memorizing idiosyncratic patterns in training data.

### Bias, fairness, and generalizability across populations

Bias remains one of the most significant ethical and technical issues confronting medical AI. Algorithmic bias arises when AI systems exhibit systematic performance differences across demographic or clinical groups, often due to unrepresentative training data or disparate healthcare access. Clinical AI models trained mostly on data from specific regions, ethnicities, or scanner types may deliver inaccurate predictions for underrepresented populations. This problem is widely recognized in healthcare AI (Ueda et al., 2024).

Bias in AI does not only produce inequities in diagnosis or treatment recommendations but may also exacerbate existing healthcare disparities if deployed without safeguards (Table 4). Broad surveys highlight the need for diverse datasets, algorithmic fairness techniques, and interdisciplinary research bridging clinical expertise and AI development to minimize bias and improve generalizability (Chinta et al., 2025). Moreover, public and advocacy groups are increasingly pressing for equity-centered AI standards to prevent deepening racial and socioeconomic disparities in healthcare outcomes as AI tools proliferate (Chen et al., 2023).

**TABLE 4:** Data, *bias, and ethical considerations in ai brain–heart research.*

Parameter	Description	Example impact	Mitigation strategies	References
Data quality	Noise, missingness, sensor drift	Reduced model robustness	Signal preprocessing, QC pipelines	(Tripathi et al., 2021)
Labeling bias	Inconsistent clinical annotations	Model mislearning	Expert adjudication, consensus labels	(Sylolypavan et al., 2023)
Algorithmic bias	Underrepresentation of subgroups	Performance disparities	Fairness audits, stratified evaluation	(Chen et al., 2023)
Privacy and consent	Sensitive physiological data	Loss of trust, legal risk	Federated learning, encryption	(Markkandan et al., 2024)
Interpretability	Black-box predictions	Low clinician adoption	Explainable AI methods	(Vani et al., 2025)

### Privacy, consent, and data governance

AI’s reliance on large volumes of sensitive personal health information raises profound privacy and consent concerns. Medical data include highly personal diagnostic results, genetic information, and longitudinal health records. Even pseudonymized datasets can be vulnerable to re-identification attacks, which threaten confidentiality. Real-world evidence shows that existing regulatory frameworks (such as HIPAA, GDPR) are not fully equipped to manage AI-driven data use, especially when data are aggregated across institutions or used in federated learning contexts (Barbaria et al., 2025; Markkandan et al., 2024; Nasir et al., 2025).

Informed consent becomes complicated when data are repurposed for secondary use, model training, or continuous learning. Consent processes must also address complexities when data are used in AI pipelines tied to remote wearables and digital phenotyping tools, where users may not fully grasp how their data contribute to automated decision systems (Andreotta et al., 2022; Moulaei et al., 2025; Sylolypavan et al., 2023). Questions of data ownership, stewardship, and governance are also central to trust and ethical deployment, demanding institutional transparency and robust administrative oversight. Additionally, explainability and accountability remain ethical priorities; AI systems that produce opaque “black-box” decisions can undermine patient autonomy, clinician trust, and legal accountability, especially when they influence critical decisions like risk stratification or therapy recommendations (Tung et al., 2025; Vani et al., 2025).

## Challenges and knowledge gaps

### Limited multimodal datasets

A central technical challenge for brain–heart neurocardiology is the relative scarcity of large, rigorously labeled multimodal datasets that combine neural signals (e.g., EEG, fMRI) with cardiovascular data (e.g., ECG, HRV) in sufficient volume and diversity (Ajijola et al., 2025; Habecker et al., 2025; Herring et al., 2025; Swenne and Shusterman, 2025). Developing AI models capable of spanning neural and cardiac signal domains requires not just more data but comprehensive multicenter, multicohort collections. These resources are essential to mitigate overfitting and ensure models are robust against institutional and demographic variability.

Even where datasets exist, the lack of standardized preprocessing, annotation, and synchronization techniques across modalities limits the development of models that can truly exploit cross-modal relationships. Strategies such as federated learning and synthetic data generation (via generative models or digital twins) may partially alleviate data scarcity, but these approaches bring their own validation and privacy challenges.

### Temporal resolution mismatches

Neural and cardiac signals are recorded at distinct temporal scales; EEG captures millisecond–level dynamics, whereas many cardiac measures aggregate over seconds or minutes. AI models must reconcile such temporal misalignment when learning interactions across modalities. Without effective time-alignment and hierarchical temporal learning architectures, models risk missing critical interaction patterns or producing spurious associations.

Research in time-series AI, including attention mechanisms and multiresolution architectures, offers pathways to address these mismatches, but real-world validation of such methods across user populations and clinical endpoints remains an open problem.

## Model interpretability versus performance trade-offs

DL models yield high predictive power but often lack interpretability, which is crucial in clinical contexts where decisions affect patient care. Clinicians are less likely to trust or adopt systems whose internal logic cannot be explained in terms consistent with physiological and clinical reasoning. Balancing model performance and interpretability is therefore a pressing research need.

Efforts such as explainable AI (XAI), feature attribution techniques, and causal reasoning frameworks aim to surface meaningful insights into model decisions (Ahmed et al., 2026; Lamens and Bajorath, 2026; Vani et al., 2025). However, achieving high interpretability without sacrificing performance remains challenging because model simplification often reduces predictive accuracy.

## Translational barriers to clinical adoption

Numerous hurdles impede the translation of AI models from research prototypes to clinical practice. These include interoperability with existing health information systems, regulatory compliance, clinician workflow integration, real-world validation, and demonstration of clinical utility in prospective settings rather than retrospective analyses alone.

Regulatory frameworks for AI-driven medical devices are still evolving, and guidelines for continuous learning systems are nascent. Prospective clinical trials, standardized evaluation benchmarks, and post-deployment monitoring will be essential to demonstrate safety, reliability, and benefit compared to current standards of care (Alelyani, 2025).

Below is suggested text you can directly integrate into the review, written to address each of the three points in a constructive, reviewer-responsive way while remaining consistent with the tone and content of the manuscript.

## Maturity of evidence and translational readiness of AI approaches

While AI-based methods for decoding brain–heart interactions show considerable promise, it is important to distinguish clearly between levels of evidentiary maturity. Much of the current literature remains at the proof-of-concept stage, demonstrating feasibility in controlled datasets or single-center cohorts, often with retrospective designs and limited external validation. A smaller subset of studies has progressed to retrospective multicohort validation, showing improved generalizability across datasets, devices, or institutions, particularly in ECG-based arrhythmia detection and HRV-derived risk stratification. In contrast, prospectively validated or clinically deployed AI tools remain relatively rare in brain–heart research, with most real-world implementations confined to single-modality cardiac applications (e.g., wearable AF detection) rather than integrated neurocardiac modeling. Explicitly framing AI studies along this experimental–translational–clinical continuum highlights both the substantial progress achieved and the gap that remains before integrated brain–heart AI systems can be routinely incorporated into clinical workflows.

## Comparative performance, inconsistencies, and methodological trade-offs

Although many AI approaches are reported with high accuracy, their performance and robustness vary substantially depending on model architecture, data modality, and clinical context. Deep learning models generally outperform traditional machine learning when trained on large, well-annotated datasets, particularly for raw ECG waveform analysis, where convolutional architectures excel at capturing subtle morphological patterns. However, these gains are not universal: in smaller datasets or multimodal settings with heterogeneous signal quality, simpler models with engineered features may perform comparably or even more robustly. Similarly, while multimodal fusion often improves predictive accuracy, results are inconsistent across studies, reflecting differences in fusion strategy, temporal alignment, and signal preprocessing. These inconsistencies underscore that no single AI methodology is universally superior; rather, model performance is tightly coupled to data quality, sample size, and the physiological question being addressed. Greater emphasis on comparative benchmarking and negative findings would strengthen the field by clarifying when added model complexity yields meaningful gains versus when it introduces instability or overfitting.

## Dataset bias, representativeness, and equity considerations

A critical limitation of current AI-based brain–heart research is the presence of demographic, geographic, and healthcare-system biases embedded within training datasets. Wearable-derived data, in particular, disproportionately represent individuals from high-income regions, younger age groups, and populations with greater access to digital health technologies, potentially limiting model generalizability. Differences in skin tone, body habitus, comorbidity burden, occupational patterns, and healthcare access can systematically influence signal quality, labeling accuracy, and downstream model performance. Moreover, healthcare-system–specific practices (such as referral patterns, monitoring intensity, or diagnostic thresholds) introduce structural biases that may be inadvertently learned by AI models. Without deliberate mitigation strategies (such as diverse cohort inclusion, fairness-aware model evaluation, and stratified performance reporting) AI systems risk reinforcing existing disparities rather than alleviating them. Explicit acknowledgment of these biases, particularly in wearable-based and population-scale studies, is essential for responsible translation of AI-driven brain–heart technologies into equitable clinical practice.

## Key unanswered questions

Despite rapid advances in AI-enabled analysis of brain–heart interactions, several fundamental questions remain unresolved. First, to what extent do current AI models capture true physiological coupling versus correlational patterns driven by shared covariates or dataset artifacts? Establishing causal directionality and mechanistic validity remains a major challenge, particularly in multimodal neural–cardiac datasets with temporal misalignment.

Second, what level of multimodal complexity is truly necessary for clinical utility? While integrated EEG–ECG and imaging-based models often outperform single-modality approaches in retrospective studies, it remains unclear whether these gains justify the added cost, data burden, and interpretability challenges in real-world clinical settings.

Third, how generalizable are AI-derived brain–heart biomarkers across populations, devices, and healthcare systems? Many models are trained on homogeneous cohorts or specific wearable platforms, raising concerns about robustness when deployed across diverse demographic groups or in low-resource settings.

Fourth, what constitutes adequate prospective validation for AI-based neurocardiac tools? Clear standards are lacking regarding trial design, performance benchmarks, and clinically meaningful endpoints, particularly for continuous monitoring and adaptive AI systems.

Finally, how can ethical, regulatory, and governance frameworks evolve to support continuous, real-world AI monitoring of brain–heart dynamics while preserving privacy, equity, and clinician trust? Addressing these questions will be essential for translating AI-driven insights from experimental and translational research into safe, effective, and equitable clinical practice.

## Future directions

Digital twin technologies (virtual replicas of physical systems updated continuously with real-world data) represent a promising architecture for brain–heart AI modeling (Cen et al., 2023; Sarris et al., 2023; Takahashi et al., 2025; Upadrista et al., 2025). Digital cardiovascular twins have been explored for predicting arrhythmia recurrence and personalizing hemodynamic modeling, suggesting utility in individualized risk assessment and therapy planning (Mehra et al., 2026; Strocchi et al., 2025; Tasmurzayev et al., 2025; Waight et al., 2025a; Waight et al., 2025b).

Extending this concept to neurocardiac twins that integrate neural activity, cardiovascular signals, genomic data, and behavioral patterns could enable mechanistic simulations of brain–heart interactions (Adnan et al., 2025). These models could forecast dynamic responses to interventions, identify early signs of dysregulation, and simulate clinical scenarios without direct patient exposure.

To enable large-scale, collaborative model training without centralized data sharing, federated learning (FL) and other privacy-preserving AI paradigms are gaining traction (Di Cosmo et al., 2025; Goto et al., 2022; Guo et al., 2026; Liu X. et al., 2025; Malpetti et al., 2025; Rahman et al., 2024; Ryu et al., 2025; Tölle et al., 2025; van Genderen et al., 2024; Weimann and Conrad, 2024; Xu et al., 2022; Yordanov et al., 2024). FL allows institutions to contribute to model improvements while keeping patient data local, reducing privacy risks. However, ethical issues such as “federation opacity” (where model behavior is influenced by unseen local biases) must be addressed through transparency and audit frameworks (Shah et al., 2025). In addition, techniques like differential privacy, secure multiparty computation, and homomorphic encryption can preserve data confidentiality during AI training and inference, offering pathways to scalable yet ethical AI solutions.

AI-driven closed-loop neuromodulation systems may harness continuous monitoring of neural and cardiac signals to guide therapeutic stimulation (e.g., vagus nerve stimulation) in real time (Huang G. et al., 2025; Mathews et al., 2025). Such systems could adapt stimulation parameters based on autonomic state or arrhythmic risk, creating personalized interventions that respond to instantaneous physiological conditions.

Advancing the clinical adoption of AI systems will require robust regulatory frameworks that account for AI’s adaptive nature, continuous learning, and decision support role. Regulatory agencies are increasingly issuing guidance for AI/ML-based medical devices, emphasizing validation on diverse cohorts, performance monitoring post-deployment, and risk management for fairness and safety (Singh et al., 2025). Prospective clinical trials designed specifically for AI tools (including randomized evaluations and real-world performance studies) will bolster evidence for efficacy and cost-effectiveness.

## Conclusion

AI’s integration into neurocardiology and healthcare more broadly has ushered in a new era of multimodal data analysis, enabling joint interpretation of neural, cardiac, and behavioral signals. Advances in ML, explainable AI, federated learning, and digital twin technologies are expanding the potential to decode complex brain–heart interactions with increasing precision and clinical relevance.

From continuous wearable monitoring to personalized digital twins and AI-guided closed-loop therapies, these technologies hold promise for early diagnosis, individualized risk stratification, and dynamic intervention strategies. Ethical frameworks, fairness-aware algorithms, and privacy-preserving designs are critical to responsible deployment and equitable benefit across diverse populations. The convergence of AI with wearable sensing and personalized modeling opens the possibility of individualized predictive neurocardiology, where continuous, real-world monitoring informs precision diagnostics and interventions. To realize this vision, robust data governance, interdisciplinary partnerships, and adaptive regulatory ecosystems are essential.

In summary, while significant challenges remain (including bias mitigation, dataset expansion, interpretability, and clinical validation) the rapid evolution of AI technologies offers a roadmap toward safer, more equitable, and scientifically grounded applications in brain–heart health. With thoughtful governance and ongoing research that centers ethical principles alongside technical innovation, AI-driven neurocardiology can transform both scientific understanding and patient care.
